# Apple polyphenols delay postharvest senescence and quality deterioration of ‘Jinshayou’ pummelo fruit during storage

**DOI:** 10.3389/fpls.2022.1117106

**Published:** 2023-01-19

**Authors:** Ya-Jie Zhang, Qiang Huang, Ao-Ran Li, Zeng-Yu Gan, Jiao-Ke Zeng, Wen-Bin Kai, Chu-Ying Chen, Jin-Yin Chen

**Affiliations:** Jiangxi Key Laboratory for Postharvest Technology and Nondestructive Testing of Fruits & Vegetables, College of Agronomy, Jiangxi Agricultural University, Nanchang, Jiangxi, China

**Keywords:** ‘Jinshayou’ pummelo, apple polyphenols, postharvest senescence, nutritional quality, antioxidant capacity, ROS-scavenging system

## Abstract

**Introduction:**

Apple polyphenols (AP), derived from the peel of mature-green apples, are widely used as natural plant-derived preservatives in the postharvest preservation of numerous horticultural products.

**Methods:**

The goal of this research was to investigate how AP (at 0.5% and 1.0%) influences senescence-related physiological parameters and antioxidant capacity of ‘Jinshayou’ pummelo fruits stored at 20°C for 90 d.

**Results:**

The treating pummelo fruit with AP could effectively retard the loss of green color and internal nutritional quality, resulting in higher levels of total soluble solid (TSS) content, titratable acidity (TA) content and pericarp firmness, thus maintaining the overall quality. Concurrently, AP treatment promoted the increases in ascorbic acid, reduced glutathione, total phenols (TP) and total flavonoids (TF) contents, increased the scavenging rates of 2,2-diphenyl-1-picryl-hydrazyl-hydrate (DPPH) and hydroxyl radical (•OH), and enhanced the activities of superoxide dismutase (SOD), catalase, peroxidase, ascorbate peroxidase (APX), and glutathione reductase (GR) as well as their encoding genes expression (*CmSOD*, *CmCAT*, *CmPOD*, *CmAPX*, and *CmGR*), reducing the increases in electrolyte leakage, malondialdehyde content and hydrogen peroxide level, resulting in lower fruit decay rate and weight loss rate. The storage quality of ‘Jinshayou’ pummelo fruit was found to be maintained best with a 1.0% AP concentration.

**Conclusion:**

AP treatment can be regarded as a promising and effective preservative of delaying quality deterioration and improving antioxidant capacity of ‘Jinshayou’ pummelo fruit during storage at room temperature.

## 1 Introduction

Pummelo (*Citrus maxima* Merr.) fruit, the largest known citrus fruit, is a non-climacteric subtropical fruit belonging to the *Citrus* family, and widely cultivated in Southeast China (e.g., Fujian, Jiangxi, Zhejiang, Guangdong, and other adjoining provinces) (Chen et al., 2021; Chen et al., 2022). Owing to its rich nutrients (e.g., dietary fiber, organic acids, vitamins, pectins, flavonoids and minerals), full succulency, attractive appearance, pleasant flavor, and blessed moral, pummelo fruit is considered as a well-welcomed citrus fruit for consumers (Nie et al., 2020; Ding et al., 2022). However, pummelo fruit severs its connection to the tree in terms of water and nutrients after harvest, and must still undergo respiration and other forms of physiological metabolisms, consuming the fruit’s own nutrients and resulting in an acute deterioration in fruit nutritional quality during post-storage (Nie et al., 2020). Due to its large size and heavy weight, ‘Jinshayou’ pummelo preservation is a crucial issue that has to be addressed. In recent years, several preservation methods have been employed to maintain postharvest storability of harvested pummelo fruit, such as 1-methylcyclopropene (Lacerna et al., 2018), CaCl_2_ (Chen et al., 2005), chitosan (Nie et al., 2020; Chen et al., 2021), and gibberellic acid (Porat et al., 2001)., As these above mentioned methods are inconvenience and unsatisfactory, there is a need for the development of effective postharvest technologies for pummelo fruit preservation.

One of the molecular mechanisms underlying fruit postharvest senescence is the induction of oxidative stress (Lum et al., 2016; Wang et al., 2021; Zhang et al., 2021; Ackah et al., 2022). Oxidative damage to horticultural fruits, such as apple, litchi, ponkan, and tomato, is reported to contribute to the imbalance of reactive oxygen species (ROS) homeostasis, potentially reducing their resistance to postharvest senescence stress (Zhang et al., 2018; Chen et al., 2021; Huang et al., 2021). As one kind of plant polyphenols, apple polyphenols (AP), extracted from the peel and pomace of green-ripened apple, is rich in antioxidants include phenolic acids, procyanidins, anthocyanins, flavonols, as well as dihydrochalcones (Rana and Bhushan, 2016; Su et al., 2019), and has gained much attention in recent years for their potential to provide a variety of health benefits, including reducing oxidative stress, inhibiting *α*-glucosidase, preventing chronic ethanol exposure-induced neural injury, as well as decreasing the risk of type II diabetes, cardiovascular and intestinal diseases (Riaz et al., 2018; Li et al., 2019; Gong et al., 2020; Wang et al., 2020). In addition to health benefits, AP as a natural antioxidant has been widely used to reduce oxidative stress *via* elevating antioxidant ability, improving ROS-scavenging system, or inhibiting lipid and protein oxidation. In earlier studies, pre-storage application of 5.0 g/L AP treatment *via* immersion means was found to be effective in alleviating postharvest pericarp browning and improving the antioxidant activity, thereby contributing to the maintenance of the edible quality and extension of shelf-life in harvested litchi fruit (Zhang et al., 2015; Su et al., 2019; Bai et al., 2022). In addition, Xiang et al. (2022) reported that AP remarkably inhibited the mycelial growth of *Peronophythora litchii* as well as spore germination under *in vitro* condition, and effectively reinforced the disease resistance against postharvest downy blight rot through the elicitation of defense response in harvested ‘Baitangying’ litchi fruit. Fan et al. (2018) reported that AP should adopt a safe approach to preserve fresh-cut red pitaya fruit for 4 d with some delaying effects on pulp color-change, softening, nutrient deterioration, and microbial growth, ultimately to get a longer shelf-life since pitaya fruit has a high commodity value mostly consumed as a fresh-cut product. These studies suggest that AP could be an effective and promising preservative for delaying postharvest senescence of horticultural fruits or vegetables. However, there is currently no experimental evidence regarding the definite mechanism by which AP alleviates postharvest senescence and modulates antioxidant response in citrus fruit. Therefore, the potential preservative effects of AP are warranting further investigation to elucidate its underlying mechanism responsible for enhancing ROS-scavenging system in ‘Jinshayou’ pummelo fruit.

Though it has been shown that AP exhibits its strong antioxidant ability, there is limited research on its efficacy as a postharvest anti-senescence treatment. This study was undertaken to determine the consequences of AP treatment on postharvest senescence and nutritional quality of ‘Jinshayou’ pummelo fruit following storage at room temperature, with a focus on fruit senescence development, oxidative stress, antioxidant capacity, and the ROS-scavenging system. This study contributes to our understanding of how AP treatment can be effectively applied for pummelo fruit preservation, and may also have implications for its wider application in other horticultural fruit.

## 2 Materials and methods

### 2.1 Pummelo fruit and treatments

Mature-green ‘Jinshayou’ pummelo (*C. maxima* Merr.) fruit were harvested from Jinggang honeydew base in Ji’shui County, Jiangxi Province (27°21′26″N and 115°9′1″E), and transferred to the Jiangxi key laboratory for postharvest technology and nondestructive testing of fruits & vegetables. A total of 900 pummelo fruits were selected for uniform size, shape and absence of mechanical damage or disease, washed with tap water, and then air-dried at room temperature overnight. The selected pummelo fruits were randomly divided into three groups, with three replicates consisting of 300 (3×100) fruits for each group, and subjected to following treatments: immersion in AP (food grade, purity greater than 75%, Yuanye Biotechnology Co., Ltd., Shanghai, China) solution at 0, 0.5, or 1.0% for 5 min at 20°C. After air-drying, the control and AP-treated fruits were individually packaged in polyethylene film bag (0.02 mm thickness) and stored at 20°C with relative humidity of 80–90% for 90 d. Fruit decay index, weight loss, peel color, albedo firmness, and other biochemical quality parameters were measured at 15 d intervals. For each sample, the juice sac tissues of 15 fruits (5 fruits per replicate) randomly taken from each group were ground into powder and then stored at -80°C condition.

### 2.2 Assessment of peel color and albedo firmness

The CIE parameters of L* (dark to light), a* (green to red), and b* (blue to yellow) on two opposite equatorial sites of ‘Jinshayou’ pummelo peel were measured directly using a CR-400 colorimeter (Minolta Co., Osaka, Japan), following a recognized method described by Mitalo et al. (2022). Citrus color index (CCI) was calculated by the following Hunter lab equation: CCI = 1000 × a*/(L* × b*).

The firmness of the albedo was assessed by a GY-4 hand-held fruit hardness tester with a 7 mm probe after removal of a 3 mm flavedo section, and the result was recorded in Newton (N).

### 2.3 Measurement of fruit decay index and weight loss

The detailed method for evaluation of fruit decay index has been previously described by Huang et al. (2021). The decay index was evaluated as the number of decayed pummelo fruit those with visible symptoms of pitted peel or pathogen growth compared to the total number of pummelo fruit, and was expressed as a percentage (%) following each storage period.

At 15-day intervals during storage at 20 ± 2°C, the weight loss (WL) rate of ‘Jinshayou’ pummelo fruit was recorded according to the method of Baswal et al. (2020). The percentage (%) of WL rate was calculated compared to the initial weight.

### 2.4 Determination of biochemical quality parameters

Total soluble solid (TSS) content in pummelo juice sac was determined with a digital saccharometer (model: RA-250WE, Atago, Japan), calibrating with deionized water before each reading, and the result was expressed as a percentage (%). Titratable acidity (TA) content was analyzed in terms of citric acid by adding 4.0 g extracted juice with two drops of 1% phenolphthalein in 40 mL of distilled water, and then titrating with 0.1 M NaOH solution, and the result was calculated based on the NaOH consumption and expressed as %.

The permeability of cell membrane was estimated by determining electrolyte leakage with a DDS-307A conductivity meter (Shanghai Rex., China) following the method of Huang et al. (2021), being reported as a percentage (%) of the initial value to the final value. Malondialdehyde (MDA) content was assayed using the TBA method as described by Bakpa et al. (2022) with a slight modification. Briefly, 2.0 g of frozen juice sac was extracted with 5 mL of 10% (*m/v*) TCA solution and centrifuged (10 000 × *g* at 4°C for 20 min). Afterwards, 2.0 mL of the supernatant was mixed by adding the same volume of 0.67% TBA (dissolved in 50 mM NaOH) solution, followed by boiling water bath for 20 min, and then quickly cooled in an ice bath. Finally, the absorbance of the supernatant was recorded at three specific wavelengths (450 nm, 532 nm, and 600 nm) using a UV-Vis spectrophotometer (model: TU-1950, Persee General Instrument Co., Ltd., Beijing, China), with the results were reported as millimole per gram (mmol/g) FW.

For hydrogen peroxide (H_2_O_2_) content assay, a total of 2.0 g frozen sample was extracted with 5 mL of pre-cooled acetone and the homogenate was centrifuged (10 000 × *g* at 4°C for 20 min) to discard the residue. H_2_O_2_ content was determined using a specific detection kit (No: BC3590, Solarbio, Beijing, China) by monitoring the absorbance at 412 nm (Ackah et al., 2022), with the results reported as micromole per gram (μmol/g) on a frozen weight (FW).

Quantitative determination of ascorbic acid (AsA) content in pummelo fruit was carried out on juice sac samples according to the 2,6-dichlorophenol–indophenol (DPIP) dye titration method described by Huang et al. (2021), with L-AsA as the standard, where the AsA content was expressed as mg of AsA equivalent per 100 g of juice sac FW.

The glutathione (GSH) content of pummelo juice sac was determined using the 5,5’- dithiobis-(2-nitrobenzoic acid) reaction method, as described by Nie et al. (2020). A total of 5.0 g juice sac was homogenized with 5 mL of 5% trichloroacetic acid (TCA) solution (containing 5 mM ethylene diamine tetracetic acid) under an ice bath condition, and then centrifuged at 12,000 × g for 20 min at 4°C. The reaction solution containing the supernatant (0.4 mL), 100 mM phosphate buffer (1.0 mL, pH 8.0), 4 mM 5,5’- dithiobis-(2-nitrobenzoic acid) (0.6 mL) was incubated at 25°C for 10 min. The GSH content reported as milligram per kilogram juice sac FW was calculated from the absorbance value measured at 412 nm according to a standard curve of 100 μM reduced glutathione.

2.0 g of pummelo juice sac were homogenized with 8 mL of 1% HCl-methanol solution, followed by an extraction step at 4°C in the dark for 20 min, and vacuum filtered to remove the pumice. Following the Folin–Ciocalteu method and the AlCl_3_ colorimetric method outlined by Nxumalo et al. (2022), both total phenolics (TP) content and total flavonoids (TF) content were measured at 760 nm and 510 nm, with gallic acid (GA) and rutin as the standard, respectively, and the results of both TP and TF content were expressed as mg equivalent per 100 g (mg/100 g) of juice sac FW.

Two different assays were applied to assess the total antioxidant capacity in the juice sac of pummelo fruit: DPPH and hydroxyl radical (**·**OH) scavenging capacity assays. Determination of DPPH scavenging capacity was performed as described in Chen et al. (2022) with minor modifications. Briefly, 100 μL of the extracted juice sample was mixed with 1.0 mL of 0.1 mM DPPH solution, and allowed the mixture to stand in darkness for 30 min at 25°C before recording the absorbance at 517 nm. Adding 100 μL of deionized water to 1.0 mL of 0.1 mM DPPH solution was done in order to control the solution. The capacity to scavenge DPPH was expressed as a percentage (%) and calculated by the following formula: (control OD_517_ − sample OD_517_)/control OD_517_ ×100.

The **·**OH scavenging capacity was assayed by referring to the salicylic acid–Fenton method, as described by Chen et al. (2022) with minor modifications. Briefly, the juice supernatant was extracted from 5.0 g of pummelo juice sac with 5 mL of 50% (*v/v*) ethanol. The reaction system consisted of 1.0 mL of juice supernatant, 1.0 mL of 9 mM ferrous sulfate solution and 1.0 mL of 8.8 mM H_2_O_2_ solution, and 1.0 mL of 9 mM salicylic acid solution (dissolved in ethanol). The absorbance of the mixture was measured at 410 nm after being held in a 37°C water bath for 20 min. The **·**OH scavenging capacity was expressed as a percentage (%) and calculated by the following formula: (control OD_410_ − sample OD_410_)/control OD_410_ ×100.

### 2.5 Extraction of ROS-scavenging enzymes and the activities determination

For the enzyme extraction and activity assay, all steps were carried out at 4°C. Crude enzyme was extracted by homogenizing 2.0 g of frozen juice sac powder with 8 mL of pre-cooled 100 mM phosphate buffer (pH 7.5, containing 5 mM DTT and 5% PVP). The centrifugation at 12 000 × *g* for 30 min removed the sediment, and the supernatant was collected for assaying ROS-scavenging enzymes [e.g., superoxide dismutase (SOD), catalase (CAT), peroxide (POD), ascorbate peroxidase (APX), and glutathione reductase (GR)] activities.

SOD (EC 1.15.1.1) activity was determined *via* a specific SOD test kit (No: BC0170, Solarbio, Beijing, China) by detecting the absorbance of the reaction system at 560 nm. SOD activity was reported as U/g, where one unit (U) of SOD activity was equal to the photochemical reduction of nitroblue tetrazolium inhibited by 50% per minute.

CAT (EC 1.11.1.6) activity was determined according to the method of Carrión-Antolí et al. (2022) in the presence of H_2_O_2_. The reaction mixture contained 2.9 mL of 15 mM H_2_O_2_ and 0.1 mL of crude enzyme, and the absorbance was recorded at 240 nm. One unit of CAT activity corresponded to a 0.01 decrease in absorbance at 240 nm per minute, and the results were expressed in terms of U/g.

The guaiacol oxidation method of Nxumalo et al. (2022) was used to monitor POD (EC 1.11.1.7) activity, with a few modifications. The addition of 0.2 mL of 0.5 M H_2_O_2_ (diluted with 50 mM phosphate buffer) to 3 mL of 25 mM guaiacol solution and 0.3 mL of crude enzyme triggered the activation of the reaction mixture for POD activity. POD activity was given in U/g, where one unit (U) of POD activity was equal to the increase in the absorbance of 1 per minute at a wavelength of 470 nm.

APX (EC 1.11.1.11) activity in pummelo juice sac was measured by our previous method, as described by Nie et al. (2020). The total volume of the reaction mixture was 3 mL, made up of 2.6 mL of 50 mM phosphate buffer (pH 7.5), 0.3 mL of 20 mM H_2_O_2_ (diluted with 50 mM phosphate buffer) and 0.1 mL of enzyme crude extract. APX activity was expressed in U/g, with one unit being defined as the decrease of 0.01 in absorbance at 290 nm over one minute.

GR (EC 1.8.1.7) activity was determined following the method of Peng et al. (2022). 30 μL of nicotinamide adenine dinucleotide phosphate was added to 3.0 mL of 100 mM phosphate buffer (pH 7.5), 0.2 mL of enzyme crude extract, and 0.1 mL of 10 mM oxidized GSH to initiate the reaction mixture (3.3 mL) for GR activity. One unit of GR activity corresponded to a decline of 0.01 in absorbance at 340 nm per min, and the results were expressed as U/g.

### 2.6 RNA extraction and RT-qPCR analysis

The expressions level of *CmSOD* (Cg7g011780), *CmCAT* (Cg3g025260), *CmPOD* (Cg2g001370), *CmAPX* (Cg6g002810), and *CmGR* (Cg5g018970) was measured according to our modified methodology (Nie et al., 2020). Total RNA was extracted from 0.5 g of frozen juice sac samples on 0, 15, 30, 45, 60, 75, and 90 d in the control and AP-treated pummelo fruit according to the cetyltrimethyl ammonium bromide (CTAB) method described by Landi et al. (2021). The first-strand cDNA synthesis and RT-qPCR analysis of ROS-scavenging enzymes encoding genes were orderly performed following the procedures described by Chen et al. (2022). The reaction conditions were 95°C pre-denaturation for 30 s, 39 cycles of 95°C for 5 s and 60°C for 30 s, 95°C hold for 15 s, 60°C for 30 s (lysis curve temperature), and 95°C extension for 5°C. The *Actin* (Cg8g022300) gene was used as the internal control gene (Nie et al., 2020). The 2^-ΔΔCt^ method was using to quantify the relative expression of *CmSOD*, *CmCAT*, *CmPOD*, *CmAPX*, and *CmGR* (Livak and Schmittgen, 2001). All primers of the above mentioned genes for RT-qPCR analysis are listed in **Supplementary Table 1**.

### 2.7 Statistical analysis

The data were processed through analysis of one-way ANOVA test with SPSS Statistics Software (20.0 versions, IBM, NY, USA). The significant differences among the AP treatment means were separated at 5% level of probability at each storage time.

## 3 Results

### 3.1 Effects of AP on peel color, albedo firmness, decay index, weight loss, TSS content and TA level

**Figure 1:**
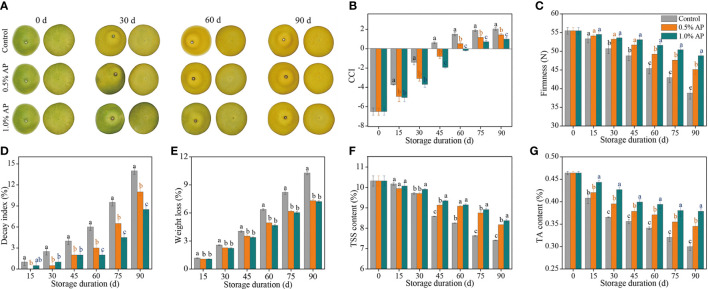
The inhibitory effects of pre-storage AP treatment on color development **(A)**, CCI **(B)**, firmness **(C)**, decay index **(D)**, weight loss **(E)**, TSS content **(F)**, and TA content **(G)** of harvested ‘Jinshayou’ pummelo fruit. The different letters for each same sampling point indicate significantly different at *P* < 0.05 according to Duncan’s multiple range test.

The color of harvested fruit is commonly used as an indicative of its appearance quality, which is why consumers use it as a criterion for procurement. The surface color of ‘Jinshayou’ pummelo fruit turns from green to yellow during storage at room temperature after harvest (**Figure 1A**). The CCI value for ‘Jinshayou’ pummelo fruit showed a gradual increase over the storage period for the non-treated (control) and two AP-treated groups (**Figure 1B**). Nevertheless, the increase in CCI value was markedly reduced by pre-storage AP treatment in comparison to the control fruit’s surface. The inhibitory effect of pre-storage AP treatment was positively correlated with the AP-treated concentration, and 1.0% AP treatment showed a significantly delayed increase in CCI value in contrast with its 0.5% concentration (**Figures 1A, B**).

The firmness of fruit is a key aspect that affects the fruit quality during postharvest storage. The albedo firmness of the control and AP-treated ‘Jinshayou’ pummelo fruit decreased throughout the storage period, reaching the lowest values at the end of storage. AP treatment at 0.5% and 1.0% effectively postponed the decline in albedo firmness, being 16.3% and 25.9% higher (*P* < 0.05) than that in the control pummelo fruit at 90 d of storage, respectively (**Figure 1C**).

As depicted in **Figure 1D**, it was observed that the decay index in the three groups continuously increased during the entire storage period. The decayed fruit began to appear after 15 d of room temperature storage, and the decay index in the control pummelo fruit reached 14.0% at 90 d of storage, while the decay index in the 0.5% and 1.0% AP-treated groups were only 11.0% and 8.5%, respectively, demonstrating that the efficacy of AP treatment on ‘Jinshayou’ pummelo fruit against postharvest senescence may be due to the defense response of AP toward abiotic stress or pathogen infection.

In general, fruit weight loss increased gradually with the extension of storage period. The weight loss of ‘Jinshayou’ pummulo fruit increased during the room temperature storage period, but the control group had a significantly higher rate increase in weight loss (*P* < 0.05; **Figure 1E**). At the end of storage period (90 d), the weight loss in the control group was 10.27%, while the weight loss in the two groups treated with 0.5% and 1.0% AP were 7.32% and 7.21%, respectively.

Both TSS and TA are known as important indicators of fruit maturity, which mainly determine the storability and overall flavor of horticultural fruits, particularly citrus fruit. TSS and TA content in pummelo juice sacs decreased in all three groups throughout the storage period, and pre-storage AP treatment notably delayed the reduction of TSS and TA contents during the room temperature storage period (**Figures 1F, G**). The contents of TSS and TA in pummelo fruit treated with 1.0% AP were 13.0% and 26.7% higher than those in the control fruit (*P* < 0.05), while both contents in the 0.5% AP-treated pummelo fruit were 10.3% and 16.7% lower than those in the control fruit at the end of the storage period (*P* < 0.05). Meanwhile, there was no significant difference in TSS content between the two AP-treated groups (**Figure 1F**). Compared to 0.5% AP treatment, the better mitigating effect for TA degradation in ‘Jinshayou’ pummulo fruit was found in the 1.0% AP treatment (**Figure 1G**).

### 3.2 Effects of AP on electrolyte leakage, MDA content and H_2_O_2_ level

**Figure 2:**
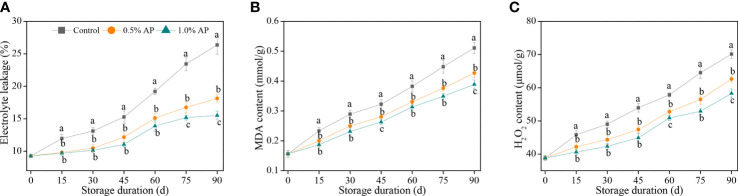
Variation in electrolyte leakage **(A)**, MDA content **(B)**, and H_2_O_2_ content **(C)** in the juice sac of ‘Jinshayou’ pummelo fruit treated without or with AP (0.5% and 1.0%) during room temperature storage period. The different letters for each same sampling point indicate significantly different at *P* < 0.05 according to Duncan’s multiple range test.

Electrolyte leakage, as a physiological marker for membrane permeability, has been widely applied to evaluate the integrity of cell membrane. As illustrated in **Figure 2A**, there was a gradual increase in electrolyte leakage of pummelo juice sacs during the storage period. A 1.0% concentration of AP resulted in a remarkable suppression of the increase in electrolyte leakage and maintained the lowest level in comparison with the control and 0.5% AP-treated groups (*P* < 0.05). The electrolyte leakage of the control pummelo fruit was 26.4% at the end of storage period, while that of the 0.5% and 1.0% AP treatments had reached 18.1% and 15.5%, respectively.

Similarly, AP treatment delayed the accumulation of MDA content compared with the control pummelo juice sacs (**Figure 2B**). The MDA content in the control pummelo juice sacs increased continuously during the storage at room temperature, while the accumulation of MDA content in both 0.5% and 1.0% AP-treated fruit showed a slower rate. The MDA content of the 1.0% AP-treated pummelo juice sacs was much lower than that of the control fruit over the whole of storage period (*P* < 0.05), suggesting that the 1.0% AP treatment may help to delay the membrane lipid peroxidation’s extent in ‘Jinshayou’ pummulo fruit under unfavorable conditions, including postharvest senescence stress.

An uptrend in the H_2_O_2_ content of each room temperature stored pummelo fruit with increasing storage period is displayed in **Figure 2C**. The H_2_O_2_ content in the juice sacs of the control group increased rapidly during storage, while both AP-treated fruit showed a remarkable lower increase of H_2_O_2_ content during the first 30 d of storage (*P* < 0.05), followed by a rapid increase. Compared with the control pummelo juice sacs, the increase in H_2_O_2_ content was decreased by 10.8% and 16.9% in the 0.5% and 1.0% AP-treated fruit at the end of storage period, respectively.

### 3.3 Effects of AP on antioxidants (AsA, GSH, phenolics and flavonoids) contents and antioxidant capacity

**Figure 3:**
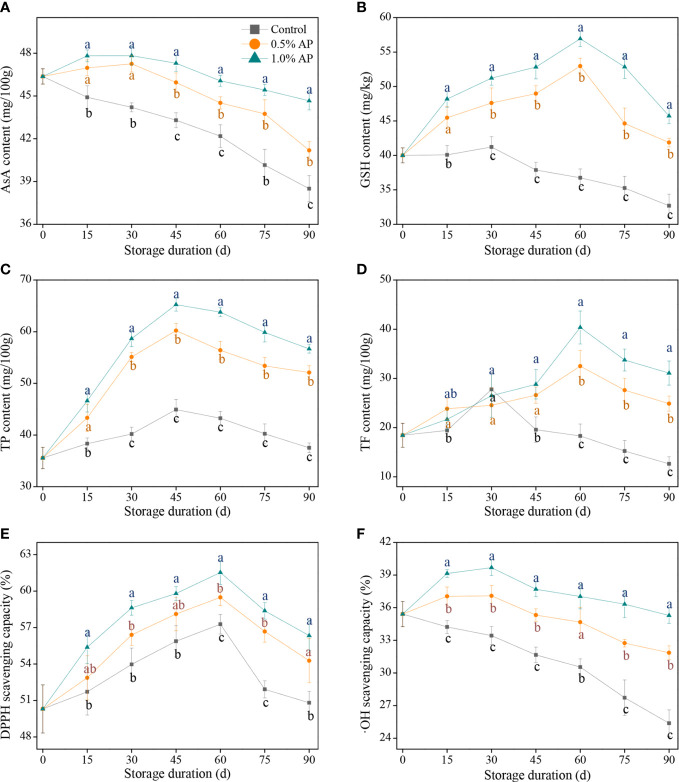
Variation in AsA content **(A)**, GSH content **(B)**, TP content **(C)**, TF content **(D)**, DPPH scavenging capacity **(E)**, and •OH scavenging capacity **(F)** in the juice sac of ‘Jinshayou’ pummelo fruit treated without or with AP (0.5% and 1.0%) during room temperature storage period. The different letters for each same sampling point indicate significantly different at *P* < 0.05 according to Duncan’s multiple range test.

AsA is not only a key primary component affecting citrus quality, but also one of the endogenous non-enzymatic antioxidants involved in the clearance of ROS over-accumulation. As illustrated in **Figure 3A**, the AsA content in pummelo juice sac from the control group decreased with storage duration, while the AsA content in 0.5% AP or 1.0% AP-treated fruits showed a slight increase during the first 30 d of storage, followed by a decline until the end of storage. Furthermore, the overall AsA content in the 1.0% AP-treated pummelo juice sac was higher than in the control and 0.5% AP-treated fruit during the middle to late stage of storage (*P* < 0.05), indicating that pre-storage 1.0% AP treatment effectively prevented the decline of AsA content, likely due to the antioxidative effect of AP.

In addition to AsA, GSH is another representative substrate in AsA-GSH system (Halliwell-Asada cycle), both of them perform a pivotal role in the AsA-GSH system together with other enzymatic antioxidant systems to maintain redox homeostasis in postharvest fruits, and their amount can directly indicate the fruits’ ability to scavenge ROS. As shown in **Figure 3B**, the GSH content in the control pummelo fruit exhibited a slight increase during the first 30 d of storage, and then decreased. By contrast, the GSH content in pummelo juice sacs treated with 0.5% AP or 1.0% AP prominently improved and peaked at 60 d of room temperature storage. The GSH content in the 1.0% AP-treated pummelo fruit was higher than that of the 0.5% AP-treated fruit (*P* < 0.05); meanwhile, in comparison with the control pummelo fruit, both 0.5% and 1.0% AP treatment led to a significant increase in GSH content, indicating that pre-storage AP treatment had a positive effect on GSH accumulation.

Phenolic compounds are a class of plant secondary metabolites that widely found in fruits, and have a variety of biological activities, particularly in relation to their antioxidant activity. The peak of TP content in each group was observed at 45 d of the room temperature storage (**Figure 3C**). The maximum TP content in the control fruit increased by 26.5% compared with the initial value (35.6 ± 2.1 mg/100g), while those in the 0.5% and 1.0% AP-treated juice sacs increased by 69.1% and 83.2%, respectively (*P* < 0.05); meanwhile, ‘Jinshayou’ pummelo fruit treated with AP at 0.5% or 1.0% had significantly higher levels of the TP content when compared to the control fruit throughout the storage period (*P* < 0.05). This evidence suggested that pre-storage AP treatment may promote the accumulation of phenolic compounds and delay their degradation in the later stage of storage.

The TF content is one of the important indexes to evaluate the antioxidant capacity of harvested fruits. The control pummelo fruit achieved its maximum TF content at 30 d of postharvest storage, and then experienced a sharp decline thereafter (**Figure 3D**). Interestingly, 0.5% or 1.0% AP treatment reached the peak of TF content until 60 d of storage and decreased gradually afterward. The TF content of 1.0% AP-treated pummelo juice sacs was significantly higher than the control and 0.5% AP-treated groups after 60, 75, and 90 d of storage (*P* < 0.05; **Figure 3D**), suggesting that 1.0% AP treatment had the most effective ability to preserve TF content.

**Figure 4:**
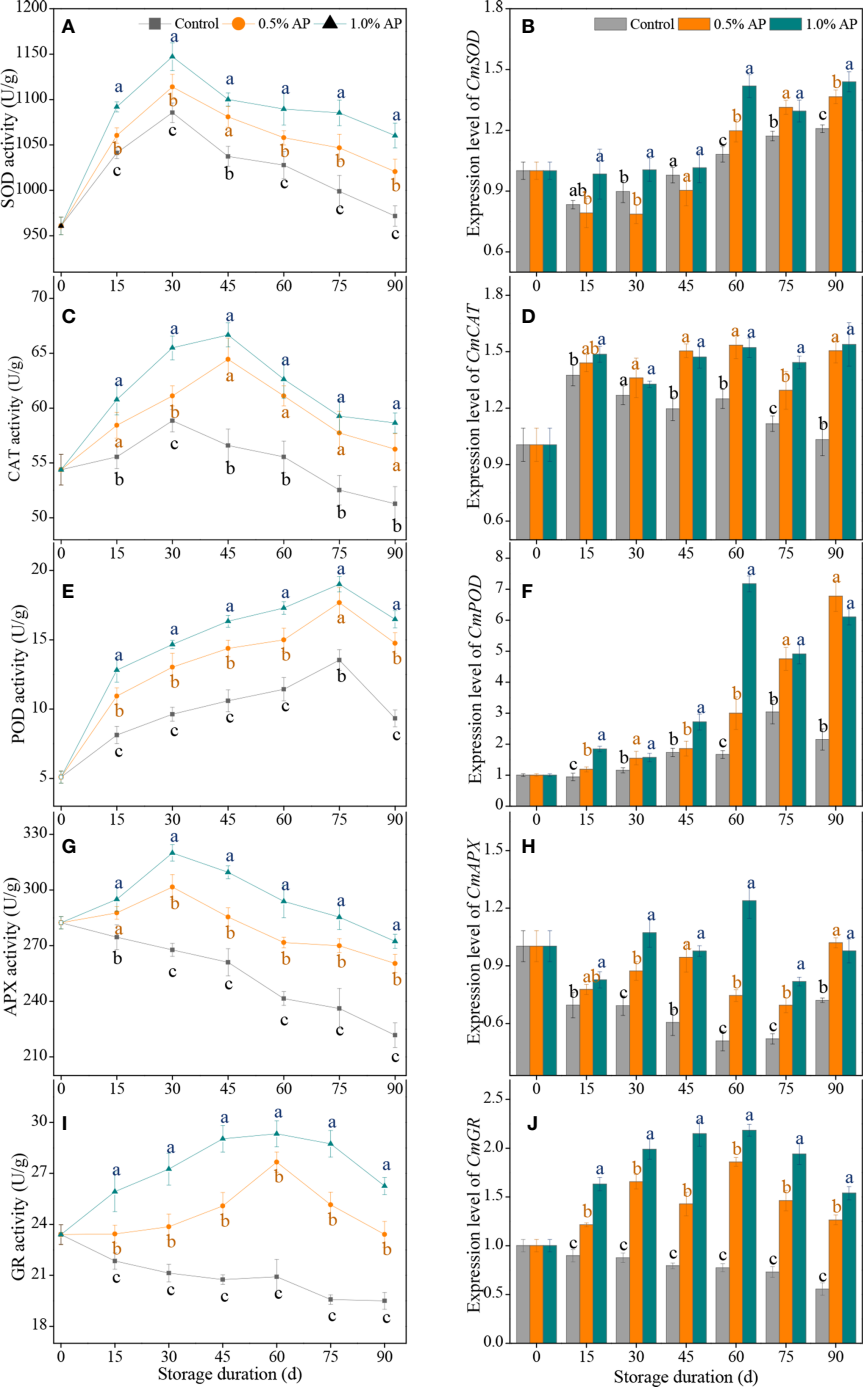
The activities and expression levels of SOD **(A, B)**, CAT **(C, D)**, POD **(E, F)**, APX **(G, H)**, and GR **(I, J)** in the juice sac of ‘Jinshayou’ pummelo fruit treated without or with AP (0.5% and 1.0%) during room temperature storage period. *β-actin* (Cg8g022300) was used as the internal control gene. The different letters for each same sampling point indicate significantly different at *P* < 0.05 according to Duncan’s multiple range test.

The DPPH scavenging capacity is a key element in evaluating fruit antioxidant activity. Similar to the overall variations of TP content, the DPPH scavenging capacity of pummelo fruit reached the peak at 60 d of storage, after which it rapidly began to decline (**Figure 4E**). Compared with the initial value (50.3 ± 2.0%) at 0 d, three increases of the highest •OH scavenging capacity in the control, 0.5% and 1.0% AP-treated pummelo juice sac were 13.9%, 18.2% and 22.3%, respectively, showing that pre-storage AP treatment could reduce the oxidative damage by enhancing the ability to scavenge ROS radicals in ‘Jinshayou’ pummelo fruit.

The •OH scavenging capacity in the control juice sacs decreased throughout the storage period, similar to AsA content. In the AP-treated pummelo fruit, the •OH scavenging capacity in the juice sacs slightly increased and peaked at 30 d of room temperature storage, followed by a progressive decline (**Figure 3F**). It is worth mentioning that the control pummelo fruit showed the lowest levels of •OH scavenging capacity over the entire storage period (*P* < 0.05), indicating that the •OH scavenging capacity in pummelo juice sacs treated with 0.5% and 1.0% AP were overall 1.14 and 1.23 times higher, respectively, compared to the control pummelo fruit.

### 3.4 Effects of AP on ROS-scavenging enzyme activities and their encoding expression levels

During the room temperature storage, the accumulation of excess ROS could lead to a decline in storage quality due to oxidative stress, as could the enzymatic antioxidant system. The activities of antioxidant enzymes, including SOD, CAT, POD, APX, and GR, were detected in this paper. The SOD activity in pummelo juice sacs showed a sharp increase in the first 30 d of storage in all three groups, and both AP-treated fruits showed a higher SOD activity compared to the control pummelo fruit (*P* < 0.05; **Figure 4A**). The expression level of *CmSOD* gene was strongly stimulated in the 1.0% AP-treated pummelo juice sacs during the last 30 d of storage (**Figure 4B**).

Compared to the control pummelo, 1.0% AP treatment significantly increased CAT activity over the storage periods, especially the peak of CAT activity at 45 d causing approximately 17.8% increase. Although 0.5% AP treatment also showed some increase in CAT activity, there was no noticeable difference between both treated groups, except that observed at 30 d (**Figure 4C**). Concurrently, both 0.5% and 1.0% AP treatments significantly increased the expression level of *CmCAT* gene after 30 d of storage, which were overall 27.0% and 29.6% higher than that in the control group, respectively (*P* < 0.05; **Figure 4D**).

The POD activity in the control, 0.5% AP and 1.0% AP-treated pummelo juice sac increased with the extension of storage duration, reaching the peak values of 13.53 ± 0.75, 17.67 ± 1.10 and 19.01 ± 0.55 U/g at 75 d, and then followed by a decline until the end of storage (**Figure 4E**). Compared to the control group, both AP-treated pummelo juice sacs exhibited significantly higher levels of POD activity throughout the room temperature storage (*P* < 0.05). Additionally, the up-regulated expression levels of *CmPOD* gene were appeared in pummelo juice sac following treatment with 1.0% AP, with a noticeable discrepancy in the whole of storage (*P* < 0.05; **Figure 4F**).

A gradual decrease in APX activity was observed in the control group (**Figure 4G**). Unlike the control, pummelo juice sacs treated with AP at 0.5% and 1.0% exhibited higher APX activity throughout the entire storage period (*P* < 0.05), resulting in an overall increase in APX activity of 11.6% and 18.4%, respectively. The expression level of *CmAPX* gene exhibited a trend similar to that of APX activity. During the late 60 d of storage period, both 0.5% and 1.0% AP treatments significantly increased the expression level of *CmAPX* gene in pummelo juice sacs (*P* < 0.05), with an overall increase in *CmAPX* expression of 40.9% and 67.2%, respectively (**Figure 4H**).

The GR activity in the control pummelo juice sac consistently decreased over the storage period, while that in both AP-treated fruit increased gradually, peaking at 60 d of storage, before decreasing in the rest of storage period (**Figure 4I**). The 1.0% AP-treated pummelo juice sac displayed a higher level of GR activity than the control and 0.5% AP-treated groups (*P* < 0.05), with a noticeable discrepancy during the whole storage period. As shown in **Figure 4J**, the expression level of *CmGR* gene in the control fruit was continuously decreased during the whole storage at room temperature, while pre-storage AP treatment resulted in a remarkable up-regulation of this gene expression throughout the storage period (*P* < 0.05).

### 3.5 Correlation analysis

**Figure 5:**
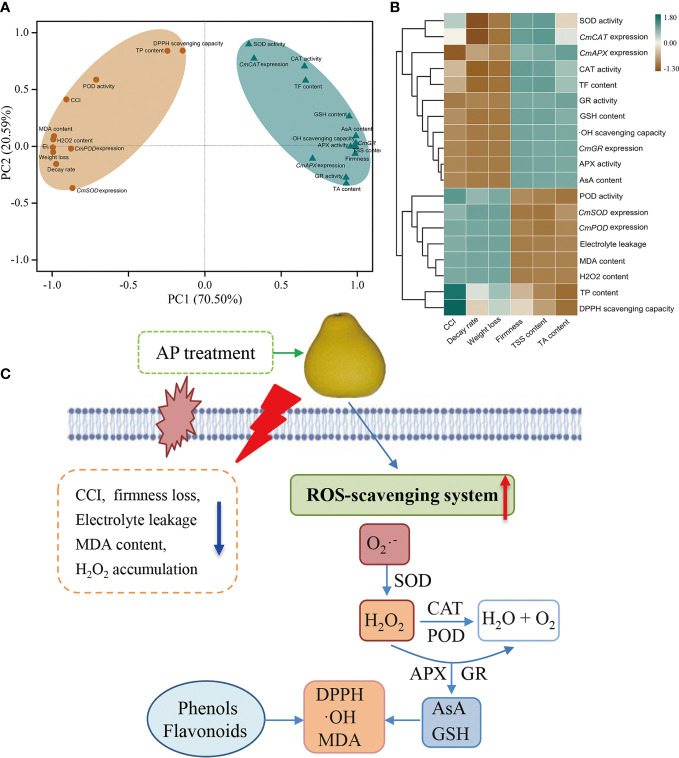
The PCA **(A)** and correlation analysis **(B)** of the ROS metabolism-related parameters in ‘Jinshayou’ pummelo fruit stored at 20°C for 90 d. A proposed model of the potential mechanism of pre-storage AP treatment delayed postharvest senescence and quality deterioration by regulating ROS-scavenging system in harvested ‘Jinshayou’ pummelo fruit **(C)**.

To understand the impact of AP treatment on postharvest senescence and quality deterioration of ‘Jinshayou’ pummelo fruit, both principal component analysis (PCA) and correlation analysis were applied to identify the ROS metabolism-related parameters measured above. All 29 parameters associated with fruit postharvest ROS metabolism were clustered into two primary categories (PC1: 70.50%; PC2: 20.59%, **Figure 5A**). The PC1 consisted of 11 parameters, including CCI, fruit decay rate, weight loss, electrolyte leakage, MDA content, H_2_O_2_ content, TP content, DPPH scavenging capacity, POD activity, *CmSOD* and *CmPOD* expression (**Figure 5A**). PC2 demonstrated a greater relationship factor for five nutritional/functional components (TSS, TA, AsA, GSH, and TF) contents, firmness, •OH scavenging capacity, four ROS-scavenging enzymes activities, as well as the expression level of *CmCAT*, *CmAPX* and *CmGR* gene, exhibiting a high negative correlation to postharvest quality deterioration (**Figure 5B**; *P* < 0.05 or 0.01). The development of fruit postharvest senescence is a result of the imbalance of ROS homeostasis, causing an increase of defective fruit as well as the degradation of fruit quality. In the present study, AP treatments had the preservative effects of reducing senescence-related indicators, such as CCI, firmness, electrolyte leakage, MDA content, and H_2_O_2_ content, while also enhancing the non-enzymatic (AsA, GSH, TP, and TF) and enzymatic (SOD, CAT, APX, and GR) ROS-scavenging systems (**Figure 5C**). These findings well demonstrated that postharvest senescence and quality deterioration in ‘Jinshayou’ pummelo fruit is accompanied by a reduction in the antioxidant system and the overall quality, thus suggesting that ROS metabolism is closely linked to postharvest storability in ‘Jinshayou’ pummelo fruit stored at room temperature.

## 4 Discussion

Pummelo fruit is the largest known citrus fruit and is extremely prone to water loss and decay rot after harvest, which seriously reduces its edible quality and commodity value. Polyphenols are important plant secondary metabolites with strong antioxidant capacity, and they have their potential to preserve fruit under postharvest conditions (Zhang et al., 2015; Su et al., 2019; Yu et al., 2021; Bai et al., 2022; Li et al., 2022; Xiang et al., 2022). AP, a natural polyphenol found in green-ripened apple, has been shown to be beneficial in inhibiting *α*-glucosidase, enhancing human immunity, deferring organism aging, preventing chronic ethanol exposure-induced neural injury, and reducing the risk of type II diabetes, cardiovascular and intestinal diseases (Riaz et al., 2018; Li et al., 2019; Gong et al., 2020; Wang et al., 2020). It also has excellent antioxidant ability to scavenge free radicals and protect plant cells from oxidative stress-caused damages (Su et al., 2019; Wang et al., 2020). Numerous studies have demonstrated that pre-storage AP treatment can retard postharvest senescence and quality deterioration of various fruits, and also improve their resistance to abiotic stress (Fan et al., 2018; Su et al., 2019; Riaz et al., 2021; Bai et al., 2022). In this study, pre-storage application of AP was found to effectively delay fruit color change and softening, as well as reducing fruit decay and weight loss of harvested ‘Jinshayou’ pummelo fruit during storage at room temperature (**Figures 1A–E**). Pre-storage AP treatment at 1.0% was more effective in terms of prolonging the storage duration to 90 d at 20°C. Nie et al. (2020) found that the 1.5% chitosan-based coating could reduce postharvest loss of ‘Majiayou’ pummelo fruit, and the storability and nutritional quality maintained for 120 d.

The levels of TSS and TA in fresh fruits correspond to the flavor and nutritional quality. As harvested fruits undergo senescence, the soluble sugar and organic acids gradually degrade; however, delaying this process can help to maintain the fruit’s flavor quality and extend its storage life (Chen et al., 2005; Baswal et al., 2020; Serna-Escolano et al., 2021). Our result regarding the downtrends of soluble sugar and organic acids in pummelo juice sacs during storage at room temperature is consistent to a previous finding of Huang et al. (2021). The data shown in **Figures 1F, G** reveals that pre-storage AP treatment at 0.5% and 1.0% can delay the declines of TSS and TA content in ‘Jinshayou’ pummelo fruit after 30 d of storage period, as both levels are higher than the control fruit, which were in accordance with pre-storage treatment of AP to ‘Dingxiang’ litchi (Su et al., 2019). Moreover, it was noted that 1.0% AP treatment displayed a more effective delay in TA degradation, from which the highest TA content was obtained during the whole storage period. Therefore, ‘Jinshayou’ pummelo fruit treated with 1.0% AP is likely to have a better storage life in terms of flavor quality compared to the control.

Postharvest senescence and quality deterioration of fresh fruit after harvest may be partially due to excess ROS-induced oxidative stress, leading to a loss of cell membrane integrity (Fan et al., 2018; Huang et al., 2021; Hanaei et al., 2022). Prolonged exposure to senescence-elicited oxidative stress results in membrane lipid peroxidation. Electrolyte leakage and MDA content are widely used to assess the integrity of cell membranes (Bakpa et al., 2022; Chen et al., 2022). In the present study, postharvest loss development in pummelo fruit stored at 20°C for 90 d may be likely due to the loss of cell membrane integrity and membrane lipid peroxidation, as revealed by the increase in electrolyte leakage, MDA accumulation and H_2_O_2_ content. Pre-storage treatment with AP at 0.5% and 1.0% resulted in lower levels of electrolyte leakage, MDA and H_2_O_2_ content, protecting cell membrane’s integrity in ‘Jinshayou’ pummelo fruit from ROS-induced oxidative damage. Pre-storage AP treatment with its antioxidant potential has been shown to be effective in reducing ROS-induced oxidative stress, as evidenced by lower levels of electrolyte leakage, MDA accumulation and H_2_O_2_ content following pre-storage AP application in ‘Dingxiang’ litchi fruit (Su et al., 2019), ‘Dongzao’ winter jujube fruit (Zhang et al., 2016), and ‘Hongyan’ strawberry fruit (Riaz et al., 2021), which has well alleviated postharvest senescence and quality deterioration in ‘Jinshayou’ pummelo fruit.

In order to reduce the accumulated ROS-caused oxidative damage and maintain the homeostasis of ROS level in plant cells, it is extremely important to motivate their ROS-scavenging system, including both the non-enzymatic scavenging system and the enzymatic scavenging system (Lum et al., 2016; Chen et al., 2022; Peng et al., 2022). AsA is considered as not only a key primary component affecting citrus quality, but also one of the endogenous non-enzymatic antioxidants in AsA-GSH system (Halliwell-Asada cycle) scavenging over-accumulated ROS in fruit (Nie et al., 2020; Serna-Escolano et al., 2021; Ackah et al., 2022). Additionally, GSH is another representative substrate in AsA-GSH system involved in the clearance of ROS over-accumulation, which maintains ROS homeostasis and delays cell senescence caused by oxidative stress (Hanaei et al., 2022; Peng et al., 2022). Phenolics compounds are a class of plant secondary metabolites that perform a pivotal role in the color conversion, flavor formation, and stress resistance of harvested fruits, in conjunction with flavonoids, which protect them from the oxidative damage caused by the over-production of ROS (Ackah et al., 2022; Jiang et al., 2022). Therefore, high levels of antioxidants (AsA, GSH, phenolics, and flavonoids) contents are closely related to the fruit’s resistance to postharvest senescence stress. Our findings from this experiment demonstrated that the 1.0% AP-treated pummelo juice sacs resulted in remarkable elevations of AsA, GSH, TP and TF contents in comparison to the control sample (**Figures 3A–D**). Correspondingly, both scavenging capacity for DPPH and •OH were maintained at the higher levels than those in the control pummelo fruit (**Figures 3E, F**). Similar improvements in antioxidants amount and antioxidant capacity were also observed in ‘Majiayou’ pummelo treated with 1.5% chitosan (Nie et al., 2020), ‘Kinnow’ mandarin treated with 2.0% gum Arabic enriched (0.5-1.0%) ZnO-NPs (Nxumalo et al., 2022), ‘Fino’ lemon treated with 0.5 mM salicylic acid (Serna-Escolano et al., 2021), ‘Rio Red’ grapefruit treated with 10 g/L pectic oligosaccharides (Vera-Guzmán et al., 2019), ‘Satsuma’ orange treated with hot (40°C) electrolyzed functional water (Shi et al., 2020), and kumquat fruit treated with 300 mg/L ellagic acid (Liu et al., 2018). Zhang et al. (2015) and Su et al. (2019) found that litchi fruit treated with 0.5% AP dipping presented the higher levels of AsA, GSH, and TP content, as well as superior DPPH scavenging capacity, which may be beneficial to delay pericarp browning and maintaining postharvest quality during the storage at 25°C. Our results in this study in combination with previous reports has well demonstrated that pre-storage AP treatment can maintain the antioxidant capacity of harvested ‘Jinshayou’ pummelo fruit by decreasing the consumption of endogenous antioxidants in the juice sacs, which in turn reduces the ROS-caused oxidative damage and delays the quality deterioration of pummelo fruit.

In addition to non-enzymatic ROS-scavenging system, the enzymatic ROS-scavenging system also has its irreplaceable role in reducing the oxidative senescence in harvested fruits (Zhang et al., 2015; Lum et al., 2016; Nie et al., 2020). During the long-term storage at 20°C, the triggering of ROS accumulation in pummelo fruit may be responsible for the oxidative damage, thereby leading to an unacceptable deterioration in fruit quality. SOD, CAT, POD, APX, and GR are the key antioxidant enzymes in the enzymatic ROS-scavenging system to delay harvested pummelo fruit senescence. For instance, SOD is the only enzyme in plant cells with the disproportionation ability to dismutate superoxide anions into H_2_O_2_ and H_2_O (Serna-Escolano et al., 2021; Mitalo et al., 2022). Then, CAT and POD are two important antioxidant enzymes that act synergistically to decompose the produced H_2_O_2_ into H_2_O and O_2_, thus reducing the harmful effect of over-produced H_2_O_2_ in plant cells (Carrión-Antolí et al., 2022; Hanaei et al., 2022; Nxumalo et al., 2022). At the same time, APX and GR, as the key enzymes in AsA-GSH system, have a crucial part in maintaining redox homeostasis and protecting plant cells form excess ROS-incited oxidative damage. It has been well demanstrated that the delay in postharvest senescence and quality deterioration is associated with the enhancement of enzymatic ROS-scavenging system (Nie et al., 2020; Ackah et al., 2022; Peng et al., 2022). In this study, the higher levels of SOD, CAT, POD, APX, and GR activities was observed in the AP-treated pummelo fruit; furthermore, the expression levels of these genes encoding *CmCAT*, *CmPOD*, *CmAPX* and *CmGR* were up-regulated by 1.0% AP treatment in pummelo fruit (**Figure 4**), accompanying by the inferior H_2_O_2_ content (**Figure 2C**), suggesting that the elimination of H_2_O_2_ in pummelo juice sacs is dependent on the improvement of SOD, CAT, POD, APX, and GR activities, and the up-regulation of their encoding gene expressions. Numerous earlier studies have illustrated that postharvest treatment with AP or other plant polyphenols (e.g., tea polyphenols, *Prunus mume* polyphenols, p-coumalic acid and procyanidins) could prompt the enzymatic ROS-scavenging system to postpone postharvest deteroration and enhance the storability of litchi (Zhang et al., 2015; Su et al., 2019; Bai et al., 2022; Xiang et al., 2022), winter jujube (Zhang et al., 2016; Yu et al., 2021), pitaya (Fan et al., 2018), strawberry (Riaz et al., 2021), banana (Chen et al., 2019), and blueberry (Mannozzi et al., 2018). All these studies suggested that the activation of enzymatic ROS-scavenging system is crucial in delaying postharvest senescence of horticultural fruits.

## 5 Conclusion

The findings of this study demonstrated that pre-storage AP treatment was effective in reducing postharvest loss of ‘Jinshayou’ pummelo fruit. This was due to 1.0% AP enhancing the activities of ROS-scavenging enzymes and up-regulating their corresponding gene expression, maintaining higher antioxidant contents as well as free radical scavenging capacity, thereby reducing oxidative stress and stabilizing the ROS homeostasis in pummelo juice sacs (**Figure 5C**). Therefore, the 1.0% AP-treated ‘Jinshayou’ pummelo fruit stored at room temperature for 90 d exhibited the decreased peel color-change, fruit softening, decay rate, and weight loss. Considering its strong antioxidant attribute and acceptable cost, AP could be an effective and safe strategy for delaying postharvest deterioration and extending the storability of harvested citrus fruit.

## Data availability statement

The original contributions presented in the study are included in the article/**Supplementary Material**. Further inquiries can be directed to the corresponding author.

## Author contributions

Y-JZ: methodology, data curation, and writing-original draft. QH: methodology, and data curation. A-RL: formal analysis, investigation, and validation. Z-YG: methodology, software, and investigation. J-KZ: software, and investigation. W-BK: investigation, and formal analysis. C-YC: conceptualization, supervision, project administration, and writing-review and editing. J-YC: conceptualization, supervision, and funding acquisition. All authors contributed to the article and approved the submitted version.
