# Supplementary material for: Apple polyphenols delay postharvest senescence and quality deterioration of ‘Jinshayou’ pummelo fruit during storage
Source: Front Plant Sci. 2023 Jan 19;13:1117106. doi: 10.3389/fpls.2022.1117106 (PMC9893410; doi:10.3389/fpls.2022.1117106)
Supplement: Supplementary file 1 [file Table_1.doc]

**Table S1** Primer sequences used in the qRT-PCR analysis.

| Gene name | Gene ID | Forward primer (5' to 3') | Reverse primer (5' to 3') |
| --- | --- | --- | --- |
| *CmSOD* | Cg7g011780 | ACTACAACAAGGCGGTCGAGC | TGAGTGGTTGACATGACCTCCG |
| *CmCAT* | Cg3g025260 | AGCCAGTTGGACGCTTGGT | TGGGGGCATTAACTGGAAGCA |
| *CmPOD* | Cg2g001370 | AGGCGTTGTTTCCTGTGCTGAT | CCTGTTTGCTGTTCTGCTGTCT |
| *CmAPX* | Cg6g002810 | CGAAATGTGCGGCGTCGG | CCCTTCGAGGCCACTCCTC |
| *CmGR* | Cg5g018970 | CCGAGCCACTACGACTTTGA | TCTTTGGCACACATCCACGA |
| *Actin* | Cg8g022300 | GCTCCAAGCAGCATGAAGATCAAGG | TGCTGGAAGGTGCTGAGGGA |
